# Treatment of Bulbar Urethral Strictures. A Review, with Personal Critical Remarks

**DOI:** 10.1100/tsw.2003.34

**Published:** 2003-05-27

**Authors:** Willem Oosterlinck

**Affiliations:** Department of Urology, University Hospital, Ghent, Belgium

**Keywords:** urethral strictures, bulbar urethra

## Abstract

This is a review article on treatment of bulbar urethral strictures with personal critical remarks on newer developments. As a treatment of first intention there exists 4 options : dilatation, urethrotomy, end to end anastomosis and free graft, open urethroplasty. Success rate of dilatation and visual urethrotomy after 4 years is only 20 en 40 % respectively. Laser urethrotomy could not fulfill expectations. End to end anastomosis obtains a very high success rate but is only applicable for short strictures. Free graft urethroplasty obtains success rates of ± 80 %. There is considerable debate on the best material for grafting. Buccal mucosa graft is the new wave, but this is not based on scientific data. Whether this graft should be used dorsally or ventrally is also a point of discussion. In view of the good results published with both techniques it is probably of no importance. Intraluminal stents are not indicated for complicated cases and give only good results in those cases which can easily be treated with other techniques. Metal self-retaining urethral stent , resorbable stents and endoscopic urethroplasty is briefly discussed. Redo’s and complicated urethral strictures need often other solutions. Here skin flap from the penile skin and scrotal flap can be used. Advantages and drawbracks of both are discussed. There is still a place for two-stage procedures in complicated redo's. The two-stage mesh-graft urethroplasty offers advantage over the use of scrotal skin. Some other rare techniques like substitution with bowel and pudendal thigh flap, to cover deep defects, are also discussed.

### INTRODUCTION

This is a review of the current concepts on the treatment of bulbar strictures with a personal critical appreciation on the newer developments. Other interesting reviews on this subject by other experts in the field, published in recent years, can be found in the reference list[1,2].

The bulbar urethra extends distally from the urogenital diaphragm up to the penoscrotal angle. Strictures of the membranous urethra, particularly after pelvic fractures, are excluded from this article. There is a major difference between the primary treatment and treatment after failure of previous open urethroplasty or in strictures complicated by periurethral abscess or extensive trauma of the genitals, such as war wounds.

### PRIMARY TREATMENT

As a treatment of first intention there exist four options: dilatation, urethrotomy, end-to-end anastomosis, and free graft, open urethroplasty.

#### Dilatation

Dilatation has been performed since ancient times and still is a traditional treatment for mild strictures. However, this treatment is rarely curative and once started, the unfortunate patient is condemned to such treatment for life. Consequently the patients are prone to false passages, bleeding, septicemia, and ultimately an impassable stricture. The considerable morbidity of life-long dilatation is well known[3]. As such, chronic dilatation can only be advocated in men with restricted life expectancy or who are unfit for surgery.

The success rate of one single dilatation was found to be approximately 20% after 4 years[4,5].

#### Urethrotomy

Traditionally, urethrotomy was blind but now is mostly done under endoscopic visual control. In spite of the initial enthusiasm for visual internal urethrotomy, the results are only slightly better than those obtained by blind dilatations.

A group of South African urologists[4,5] made a prospective randomized trial between dilatation and internal urethrotomy with a group of 100 patients in each treatment. After 4 years, the trend for urethrotomy was better, but statistical significance was not reached. The overall success rate for internal urethrotomy was 40% while it was 20% for dilatation. An earlier retrospective study in the Mayo Clinic between 1976 and 1999[6] could not find a difference between the results of both techniques in terms of the probability of not requiring retreatment within 3 years. The recurrence rate after internal urethrotomy or dilatation is lower in single, short (less than 2 cm), and bulbar strictures. The risk for recurrence is higher for penile stricture, long strictures, and those with evident periurethral scarring[7]. Nearly 60% of the strictures recur within the first year after treatment. It was the merit of Steenkamp et al.[4] to demonstrate in a prospective trial that the time to recurrence was a major prognostic factor. When stricture recurs after 3 months, a second urethrotomy is of limited value and a third one is of no value at all. On the contrary, when the patient is still stricture free after 3 months, 50% will remain stricture free up to 4 years.

### Laser Urethrotomy

After initial enthusiasm about this expensive new technology, it becomes obvious, with growing experience, that it can not fulfill expectations[8]. From the theoretical point of view, this could be expected because although laser evaporization can remove the scar tissue of the stricture, without provoking too much reaction in the depth, the epithelium has to grow up from both sides of the stricture. When the distance is too great, granular tissue will fill the gap before the epithelium grows up with a new stricture as a result[9].

### End-to-End Anastomosis

Undoubtedly this is a treatment followed with the highest success rate provided it is used in the correct indication and thus in a short stricture. Several reports confirm a success rate of about 95%[10,11,12,13].

How long the stricture may be is badly defined. In Santucci's paper the mean length was 1.7 cm, while the longest stricture was 4.5 cm. But the largest majority of the treated strictures were below 2 cm[11]. The exception on this rule can be the patient with an unusually long perineum and penis in whom dissection of the urethra from its bed over a longer distance is possible. It is our attitude that the dissection of the urethra be stopped at the penoscrotal angle in order to avoid ventral chordee formation. However, in an impotent man, in whom bowing of the penis in erection is not a problem, further dissection can be allowed.

Complications such as abscesses, ejaculatory disturbances, or sacculations are very rare after this intervention and the recurrence rate at the same place is low probably because no foreign material is used. Therefore this type of intervention can be advocated as a very attractive alternative for repeated internal urethrotomy that must be seen rather as a temporary palliation than a real cure of the stricture.

A trick to extend the possibilities to do an end-to-end anastomosis was recently brought to attention by Guralnick and Webster[14]. They include a flap graft onlay at the roof of the anastomosed urethra so that spatulation of the urethra can be avoided, gaining some length.

In case of a very short stricture, it is best to make only a longitudinal ventral incision with a transverse closure to overcome the stricture. This is very simple and rapidly done.

While considerable length can be gained in end-to-end anastomosis at the level of the membranous urethra, especially after pelvic fracture, this is rarely applicable in bulbar stricture. Indeed in the case of the membranous urethra, urethral length can be gained by making the course of the urethra shorter, by cleavage of the intracorporeal septum. In bulbar stricture, the anastomosis is before or at the beginning of the curve so that cleavage is rarely helpful.

### Free Graft Urethroplasty

Bulbar strictures requiring urethroplasty, but too long for excision and end-to-end anastomosis, are best treated by a substitution urethroplasty technique in which the stricture is opened throughout its length and the patch of suitable material inserted to restore the caliber of the urethra. There is extensive experience with this type of technique showing that the overall success rate is approximately 80%[15,16]. Nevertheless, one has to admit that whatever the material used, there is an attrition rate, although not necessarily at the side of the original operation. The question of which material is best to use has provoked considerable debate.

Experience has shown that only full-thickness skin grafts of penile skin faired well. Split-thickness skin grafts have a much higher tendency to contract. Extra genital skin has a poor taking of the graft, because it tends to be thicker than penile skin, and therefore no longer has a place in urethroplasty[17].

#### *Buccal Mucosa Graft*

This graft was developed to replace spinal skin when it was no longer available but now its use has been extended by several urologists to all free graft bulbar stricture repairs[18,19,20,21,22,23,24]. Although we consider buccal mucosa an ideal alternative, there are no clinical or experimental data indicating that a buccal mucosa graft is better than penile skin. Experiments with pigs[25] demonstrated that buccal mucosa was better than retroauricular skin and in dogs[26], less hairy abdominal skin was also worse than buccal mucosa. From these experiments, we can conclude that there is no more place for extra genital skin as we know already from previous clinical results[17]. No randomized comparative clinical trials have been performed to prove its superiority.

The results give no proof for the so-called better resistance to infection for buccal mucosa compared to penile skin. So personally, I continue to use buccal mucosa only in those cases of shortness in penile skin. Indeed penile skin can be easily harvested within the same operating field and does not need any experience of surgery in the mouth. The drawbacks of an extensive wound, e.g., 2 cm large, are completely neglected in the literature. This may provoke considerable discomfort for the patient and in one case, gave us clear inversion of the lip after taking a graft 8 cm long and 2 cm large at the underlip.

#### *Penile Skin*

Penile skin is amply present in the operating field in uncircumcised men. The circumference of the penis in adults is 10 to 12 cm and a graft taken in a spiraloid way can extend the graft up to double the size. Personally, we made grafts up to 22 cm. The dorsum penis is also good material when preputial skin is no longer available.

### Dorsal or Ventral Onlay?

In 1996, Barbagli described a different way of performing free patch urethroplasty[27]. He opened the urethral stricture at the dorsal site, after dissection of the urethra from its bed on the corpora cavernosa, instead of the ventral face of the bulbus. The flap is not sutured to the open urethra alone but also with the same stitch upon the corpora cavernosa. As such, the urethra is held open and the graft is quite immobile upon the corpora cavernosa. This technique avoids sacculation of the urethra, with ejaculatory disturbances and postvoiding dribbling. Since the first description of the technique, many authors have published their results, which were uniformly good. The technique received the support of experts in the field[28].

Mundy[29] reported on 29 patients treated with ventral onlay and 42 with dorsal urethroplasty. Of those, 3 (5%) operated with the Barbagli technique developed restricture and 4 (14%) with the ventral onlay. This was, however, not a prospective randomized study and the numbers are insufficient to draw conclusions. The figures in his article are also somewhat misleading. The spongious tissue in the bulbar region is often very thick and nearly always one can cover a graft very easily with well-vascularized spongious tissue up to the penoscrotal angle.

In my view the dorsal onlay makes the operation somewhat more difficult, certainly in inexperienced hands. Additionally, it offers a solution to sacculations, which are a rare event when the graft is well adapted and covered. It is also essential in a ventral onlay to place a large caliber urethral catheter (20 to 22 Fr) to expand the patch, in order that it remains in good contact with the supporting tissues. As a most important advantage of the Barbagli technique, I personally see the good immobilization of the graft because it is sutured to the completely immobile corpora cavernosa. However, the tunica albuginea of the corpora cavernosa is badly vascularized and this makes taking of the graft questionable. In any case, the good results published seem to contradict this theoretical fear.

About 30 years ago, a Belgian urologist working in Congo (Monseur) published a very similar technique in which he opened the urethral stricture dorsally and sutured the opened urethra, without interposition of any graft directly to the corpora cavernosa[30,31]. This was later confirmed by several publications in France[32]. This technique became forgotten not only because the technique was published in French journals, but also because the easy free graft technique by Devine and Horton became more popular in the same period. So in conclusion, it may be worthwhile to go back to this old technique as it avoids the harvesting of any graft.

### Intraluminal Stents

Metal endoprosthesis became available in 1988 and was immediately followed by a series of publications about its use. Despite reports of good success and the easy removal of it once necessary, several severe problems were noticed requiring complete removal of the urethra on the entire length of the stent[33]. Wilson et al.[34] described ten cases of stent migration and recurrent stenosis following Urolume stents. Tandem stents for long strictures are particularly associated with the development of urethral obstruction.

If one looks critically through the literature, which reports good results, it becomes obvious that primarily short strictures (less than 3 cm) have been treated successfully[35]. For this type of urethral stricture, there exists several other cheap and efficient methods for treatment. From the literature it becomes evident that stents are not indicated to solve complicated and long urethral strictures. Milroy, the pioneer of this technique, reported 50% failure rates when stents were used after urethroplasty failures[36]. We also agree with Milroy that post-traumatic urethral strictures, longer than 2 cm, may not be suitable for this technique.

### Metal Self-Retaining Urethral Stent

Yachia used a completely different principle for keeping the urethra open[37]. He used a flexible spiraloid metal stent that is completely closed. So that granular tissue cannot grow into the lumen, he leaves the stent for a year. This avoids scar retraction of the lumen of the urethra in a mechanical way. In accordance with data on wound healing and experimental data on splinting of wounds, this approach seems logical. Until now, however, Yachia and co-workers are the only ones to report their results. Certainly more experience is needed before this procedure can be advocated.

### Resorbable Stents

Closed spiraloid resorbable stents made of polyglycolic acid are also in clinical research[38]. They use the same principle: a closed stent to avoid growth of granular tissue into the lumen and avoid wound contraction of the urethra. As it is made of biodegradable material, the stent must not be removed and disappears spontaneously after months.

### Endoscopic Urethroplasty with Free Graft

The idea of endoscopic urethroplasty is not new. Several authors tried several types of techniques[39]. The attempt was made by myself[40] with a free graft around a biodegradable polyglycolic acid spiral stent which was brought at the urethral stricture after endoscopic incision. The first experience was good with only two failures out of ten. The following five patients were less successful with three failures. Whether this was due to the fact that the stent was changed or to coincidence is unclear. In the last five cases, the stent was given the capacity of more rapid extension so that it was self-retaining after application and fixation from outside was no longer needed. However the company making the stents lost interest in further development in this technique and therefore the experiment was stopped.

### Does Previous Urethrotomy Influence the Outcome of Urethroplasty?

Urethroplasty is often a second type of treatment after previous dilatation or urethrotomy. Until now, previous urethrotomy was not found to be a compromising factor for the outcome of the following urethroplasty. Recent literature further confirms this[41,42], but an evolution from a rather short stricture to a much larger and complicated stricture by repeated urethrotomy or dilatation is regularly noticed.

### When are Free Grafts not Suitable?

While grafts give excellent results when used as onlay patches, this is not the case when a complete circumference of the urethra must be replaced. It was our own experience that the failure rate went up to 35% and several other authors have confirmed this[1,15]. More recently it was demonstrated, in an experimental setting in dogs, that tubes do significantly worse than onlay patches[26].

Free grafts also give much more complications when applied near to the urethral meatus. Poor quality of the covering tissue and the easy introduction of infection in the early days after the surgical procedure probably are responsible for this phenomenon.

Other important factors for success are a good immobilization of the graft during the first 4 days, during taking of the graft, and absence of infection. Immunosuppressive treatments such as cortisteroïds are also responsible for less change of taking of the graft.

## REDO'S AND COMPLICATED URETHRAL STRICTURES

Many failures still can be treated by a second chance urethroplasty with a free graft, provided the graft is surrounded by vascularized tissue and can be placed in sterile conditions. There is, however, no chance for survival or free graft in infected and sclerotic surrounding tissue.

A second end-to-end anastomosis becomes much more questionable as it is impossible to mobilize and elongate the urethra twice. The most important reasons for failure of the first end-to-end anastomosis are either the heavy traction between both ends or insufficient resection of scar tissue at both ends at the first intervention.

Data on the success rate of internal urethrotomy of very short strictures after urethrotomy are not available, but it seems worthwhile technique to try in view of the minor invasiveness of this procedure.

An end-to-end procedure after failure of a patch urethroplasty still remains possible provided it is a short stricture and the scar tissue is not too extensive. Recurrences are regularly at other places of the urethra and can be much shorter than the initial stricture.

### COMPLICATED URETHRAL STRICTURE

When the stricture is not restricted to the bulbar urethra alone, a combined reconstruction of bulbar and penile urethra is necessary. In this case we prefer vascularized flaps to the anterior urethra and free grafts for the bulbar urethra.

Flaps bring their own vascularization and do not depend on surrounding tissues for their survival and defense against infection. So they can be used in worse conditions than free grafts. They can be used as a patch as well as a tube and are as such indicated whenever the total circumference of the urethra must be replaced. The application, however, is more complicated and requires more experience than the use of grafts. Therefore, they are less indicated as a first choice for simple urethral strictures.

#### Penile Skin Flaps

For free grafts, penile skin is a good substitute for the urethra. It is hairless, elastic, and has a good vascular pedicle from the base of the penis allowing its mobilization, deep in the perineum. This skin can easily be adapted to the roof of the urethra or around the catheter for complete reconstruction of the urethra.

A transverse island or distal penile skin flap can be isolated on a subcutaneous vascular pedicle, which when pivoted at the base of the penis can be mobilized to the bulbar urethra. Depending on the length of the penis, this patch or tube can even be mobilized up to the membranous urethra for the treatment of obstructions after pelvic fracture. When a transverse island flap is not long enough, a hockey stick extension (or so-called Q-flap[43]) can be used to treat long strictures. Transverse island flaps up to 8 cm can be dissected in nearly every penis but every distance over 10 cm becomes very questionable. In this circumstance, the Q-flap is a welcome alternative.

During the dissection of the penile flap, it is extremely important to dissect immediately deep enough from the first circumferential incision up to the Bucks fascia. Indeed, there is an avascular plane just above the Bucks fascia that can be easily dissected without traumatizing any blood vessel up to the base of the penis. Not following this avascular plane inevitably provokes damage to the subcutaneous vascularization.

The dissection of penile skin from its subcutaneous tissue provokes very thin skin on which it relies for its vascular supply, i.e., on the intradermal plexus that is very thin with vulnerable arteries. Necrosis of the most distal parts of this penile skin occurs regularly but it heals rather well per secundam.

The vascular pattern of penile skin may be altered by previous operations. This makes the ventral face of the penis often completely unsusceptible for taking flaps. Also a transversal incision of the skin (for instance a circumcision line) must be taken into account because this transverse incision has provoked interruption of vascularization at the incision lines.

### Scrotal Flaps

After Johanson's popularization of the two-stage technique and the various variations of the technique to treat bulbar strictures, the use of scrotal flaps for one-stage reconstruction became a common operation. Later on, many of the indications for the use of scrotal flaps were taken over by the free grafting techniques.

Scrotal flaps have several drawbacks. The skin may be very hairy, rather thick, and often difficult to adapt to the lumen of the urethra because of its tendency to contract during manipulation. This provokes hairballs, lithiasis, sacculations with dribbling after micturition, and ejaculatory disturbances. Therefore scrotal skin should be used only when other possibilities no longer exist. This occurs only in complicated redos.

Conversely, there exist major differences among the scrotum of patients. The skin may sometimes be nearly hairfree and the scrotum may be rather thin and smooth. If the hairs on the scrotum are pigmented (brown or black) desepilation can be undertaken before intervention by laser irradiation. Desepilation of hairballs can also be done after the operation by 50% detected depilatory cream instillation in the urethra at regular intervals[44].

Recently Gil-Vernet[45] published a nice series of 37 cases of complicated urethral strictures treated with scrotal flaps. We also continued to use it in this indication.

### Two-Stage Procedures

With current techniques, only a small percentage of patients will require two-stage reconstructive techniques. The increased hospitalization time, absence from employment, and the cost and morbidity of multiple anesthesia and procedures are disadvantages of the multistage approaches.

Nevertheless, two-stage reconstruction represents an important option in a small percentage of patients in whom single-stage substitution is not feasible; for example, for the lack of healthy penile skin for peduculated flap repair. Although it is easy to preach that all patients requiring urethral reconstructions should be referred to reference centers where more diverse reconstructive surgical experience is available, it is not feasible for many patients to travel long distances for the surgery and the common urologist must be able to apply stage urethroplasties in complex situations when necessary.

Some elderly or infirm patients may be quite accepting of perineal voiding and thus may be better served by a perineal urethrostomy than with another procedure with some risk of failure. Some patients indeed do not return for the offer of a second-stage reconstruction, finding it quite acceptable to void from the perineal stoma.

All two-stage procedures have a common principle: marsupialization of the opened urethra to healthy skin from scrotum, followed by closure of this opening to tubularization of the transport and local skin urethral tissues in a second stage. Several variations have been described which differ in regard to the choice of the penoscrotal tissue used for substitution and the way in which flaps from this area are created. Recently Johanson's urethroplasty was revised by a publication of 68 complex urethral stricture patients[46].

### Two-Stage Mesh Graft Urethroplasty

It was Schreiter who developed this technique that creates an ample amount of tissue along the opened urethra. With this technique, a hairless neourethra can be created avoiding all the drawbacks of scrotal skin[47].

The principle of the operation is the free transplantation of a full-thickness skin graft from the inner face of the thigh, which is meshed with the usual skin mesher. This meshed skin graft is transplanted along the incised longitudinal stricture of the urethra. After the complete epithelialization of the free mesh graft transplant, a new urethra is formed in a second stage as described in the other two-stage procedures.

Mesh graft transplants tend to shrink considerably. This is dependent on the thickness of the graft. Therefore the split thickness skin graft should not be cut too thick. If characters on the underlying surface can be read through the split thickness graft, the correct thickness is achieved.

A correct dressing is essential for the successful outcome of the procedure. Movements between the mesh graft and the underlying surface must be avoided under all circumstances. To achieve this, fatty gauze keeps the mesh graft dry and applies a gentle pressure on the mesh graft to enhance contact with the underlying surface.

### Two-Stage Buccal Mucosa Graft Urethroplasty

Recently, buccal mucosa graft was used to replace the dorsal wall of the urethra to close perineostomy at the second stage[48]. This new technique, which also avoids hair growth and sacculation formation, seems to be worthy of consideration.

### Substitution of the Urethra with Bowel

Substitution of the urethra in complete urethral strictures with bowel segment was described many years ago[49]. More recently, the use of a pediculated appendix graft was described[50]. In my personal view, there are sufficient techniques available to cure most of the cases, but there may be some rare cases for which you may prefer such a type of construction.

### Reconstruction of the Posterior Urethra Together with Large and Deep Perineal Defects

This remains an important surgical challenge. In our region, this situation develops mostly in paraplegic males, although severe perineal trauma can provoke similar conditions. In addition to reconstructing the urethra, one has to cover and support the newly formed urethra. The soft tissue defects of the perineal region must be filled up with well-vascularized and sufficiently bulky tissue. For filling the dead space and skin defects in the perineal region, the musculocutaneous gracilis flap has been most widely used. We described the use of the pudendal thigh fasciocutaneous flap as an easier solution to this problem. The pudendal thigh flap is available in the operating field of the urologist. The technique is relatively simple, not time consuming and causes little blood loss[51].

### SUCCESS CRITERIA

There is a lack of standards for reporting failures and successes after urethral stricture treatment that has made direct comparison of the results of different studies of questionable value. Outcome studies are usually based on radiological findings, uroflowmetry, the need for postoperative instrumentation, and the lack of complications seen, i.e., dribbling, incontinence, infection, lithiasis, and sexual function. Comparison of different techniques based on randomized prospective studies has never been done and probably will never be performed. It will be nearly impossible to find a sufficient number of comparable cases. Therefore, the recommendations found in the literature are nearly always influenced by the author's preference rather than by objective criteria.

Urethroscopy is considered by most experts as the most objective way to judge the outcome of urethral stricture repair but this has been only rarely performed until now.

Patients who evaluate their own situation after urethroplasty often do better than physicians. This was recently well demonstrated by the group of Schrieter[52] in which 24 out of 30 objective failures were satisfied with the outcome of urethral surgery. Late recurrences after many years remain a reality. Even though many years may have passed without major difficulties, surgery may still be indicated. In any case, the success rate of urethroplasty remains much higher than after urethrotomy.

### CONCLUSION

The choice of technique for urethral stricture repair depends on its length, place, previous operations, presence of scar tissue, infection, and the situation of skin in the genital area. No technique is able to cope with all situations and a wide variety of possibilities must be mastered to be able to find the best solution for each condition. Whatever the technique chosen, one has to rely on the anatomical situation and the principles of wound healing and tissue transfer. When this is not taken into account it will result in many failures[2].

Urethral surgery continues to evolve as new techniques are developed. However, new does not always mean better. This paper provides an update and review of the several surgical options in the treatment of urethral strictures.
